# Screening for novel protein targets of indomethacin in HCT116 human colon cancer cells using proteomics

**DOI:** 10.3892/ol.2013.1560

**Published:** 2013-09-03

**Authors:** YAN-LI CHENG, GUI-YING ZHANG, CUI LI, JING LIN

**Affiliations:** 1 Department of Gastroenterology, The First Hospital of Tsinghua University, Beijing 100016, P.R. China; 2 Department of Gastroenterology, Xiangya Hospital, Central South University, Changsha, Hunan 410008, P.R. China; 3 Medical Research Center, Xiangya Hospital, Central South University, Changsha, Hunan 410008, P.R. China

**Keywords:** indomethacin, 2-DE, MALDI-TOF-MS, proteome, colorectal cancer

## Abstract

Non-steroidal anti-inflammatory drugs, such as indomethacin (IN), inhibit colorectal cancer (CRC) growth through cyclooxygenase (COX)-independent mechanisms, however, the precise biological mechanisms are not completely understood. The aim of the present study was to investigate new molecular factors potentially associated with IN in HCT116 human CRC cells, which do not express COX, using a proteomic approach. The total proteins from the IN-treated and untreated groups were separated by immobilized pH gradient-based two-dimensional gel electrophoresis. The differentially-expressed proteins were identified by peptide mass fingerprint (PMF) based on matrix-assisted laser desorption/ionization time of flight mass spectrometry. The PMF maps were searched in the SWISS-PROT/TrEMBL database using the PeptIdent software. Between the IN-treated and untreated groups, a total of 45 differential protein spots were detected and 15 differentially-expressed proteins were identified by PMF. IN downregulated Wnt1-inducible signaling pathway protein 1, Bcl-2-related protein A1 and mitogen-activated protein kinase, inhibited HCT116 cell growth and induced apoptosis. In conclusion, IN may exert its effects on CRC to induce HCT116 cell apoptosis and suppress growth through COX-independent pathways.

## Introduction

Colorectal cancer (CRC) is one of the most common types of malignant tumor worldwide. In recent years, due to the early diagnosis and treatment of CRC, the mortality rates have decreased, however, the prognosis remains poor. Therefore, it is crucial to identify an effective means for the chemoprevention of CRC to reduce morbidity and mortality. Indomethacin (IN) belongs to a group of non-steroidal anti-inflammatory drugs (NSAIDs), which are associated with the decreased incidence of CRC and familial adenomatous polyposis tumor formation (1,2). Previously, it has also been shown that these drugs may inhibit cell proliferation of esophageal, stomach, liver, pancreatic and other types of cancer (3–6). In addition, previous studies have reported a 40–50% decrease in mortality from CRC with the prolonged use of NSAIDs (7,8). The well-documented pharmacological action of NSAIDs is the inhibition of cyclooxygenase (COX)-2 (9–11), but NSAIDs in general have numerous targets, other than COX-2, which may inhibit tumor cell growth and induce apoptosis, including activation of the transcription factor nuclear factor κB and extracellular signal-regulated kinase (ERK)1/2 and reactive oxygen species (ROS) generation (12–14). However, the molecular mechanisms by which IN exerts its effects are not well understood. Functional proteomics provide a high-throughput method to study the complexity of life. Proteome technology is a useful tool for the identification of new cancer markers and treatment-related changes in cancer. The comparative analysis of protein alterations between pre-treated and treated cells or tissues using high-throughput proteome technology has allowed for the identification of special treatment-related proteins and the development of new molecular-based therapies. In the current study, the total proteins from IN-treated and untreated groups were separated by immobilized pH gradient-based two-dimensional gel electrophoresis (2-DE). The various expression proteins were identified by peptide mass fingerprint (PMF), based on matrix-assisted laser desorption/ionization time of flight mass spectrometry (MALDI-TOF-MS). The PMF maps were searched in the SWISS-PROT/TrEMBL database using PeptIdent software (http://www.expasy.ch/sprot). The purpose of the current study was to use functional proteomics to identify the correlated proteins of IN-treated CRC via the non-COX-dependent pathway.

## Materials and methods

### Cell culture and materials

The HCT116 human CRC cell line was purchased from the American Type Culture Collection (ATCC, Manassas, VA, USA). The cells were cultured in RPMI-1640 medium supplemented with 10% fetal calf serum in a 37°C, 5% CO_2_ environment.

IN, thiourea, iodoacetamide, dithiothreitol (DTT) and the second-dimension sodium dodecyl sulfate (SDS)-PAGE standard proteins, TPCK-Trypsin, K_3_Fe(CN)_6_, trifluoroacetic acid (TFA), α-cyano-4-hydroxycinnamic acid (CCA), DTT and acetonitrile (ACN), were obtained from Sigma-Aldrich (St. Louis, MO, USA). RPMI-1640 and 10% FBS were purchased from the Medical College of Xiangya (Changsha, China). The BCA Protein Assay kit, Immobiline pH-gradient DryStrips (pH 3–10; 24 cm), Acrylamide, SDS, Tris, 3-[(3-cholamidopropyl)dimethylammonio]-1-propanesulfonate (CHAPS), IPGphor isoelectric focusing (IPG-IEF) cell, ImageMaster 2D Elite 4.01 analysis software and LabScan software on Imagescanner were obtained from Amersham Pharmacia Biotech (Amersham, UK). The ProTEAN II electrophoresis apparatus was obtained from Bio-Rad (Hercules, CA, USA) and the Applied Biosystems Voyager System 4307 MALDI-TOF-MS was bought from Applied Biosystems Inc. (Foster City, CA, USA).

### Sample preparation

HCT116 cells were seeded in 75-cm^2^ tissue culture flasks and grown for 1–2 days prior to use. When 50% confluent growth had been reached, the media was changed to fresh standard media or media containing 316 μmol IN (IC_50_) (15). The cells were harvested 48 h after treatment, rinsed with PBS (0.8 g/l NaCl, 0.2 g/l KCl, 1.44 g/l NaH_2_PO_4_ and 0.24 g/l KH_2_PO_4_) and trypsinized with a solution of 2.5 g/l trypsin and 0.2 g/l EDTA. After 1 min, media containing FBS was added to terminate the action of the trypsin. The resulting suspension was centrifuged at 1,000 rpm for 7 min at 4°C and the supernatant was discarded. Next, the cells were resuspended in ice-cold PBS and centrifuged at 1,500 rpm for 10 min at 4°C and the supernatant was removed. This wash step was repeated three times and the cells were stored at −80°C until further use. Protein extraction from the untreated and IN-treated cells was performed with lysis buffer. The harvested IN-treated and untreated HCT116 cells were left in lysis buffer (7 mol/l urea, 2 mol/l thiourea, 4% CHAPS, 40 mmol/l Tris and 1 mmol/l PMSF) for 30 min in ice. The resulting cell lysate was then vortexed and the sample was incubated at room temperature for 30 min. Following centrifugation at 15,000 rpm at 4°C for removal of particulate material, the protein solution was collected and stored at −80°C until use. Protein concentrations were determined using the Bio-Rad Protein Assay kit (Bio-Rad) with BSA (Sigma-Aldrich) as the standard.

### IPG-2D-PAGE

2-DE was performed mainly according to the laboratory instructions (15). IPG-IEF was run on an IEF system (Amersham Pharmacia Biotech). Immobilized pH gradient strips (24 cm long; pH 3–10) were rehydrated for 14 h with 450 μl of 2-D solubilizing solution (8 mol/l urea, 2% CHAPS, 0.5% IPG buffer, pH 3–10L, 3% DTT and a trace of bromophenol blue) containing 260 μg total proteins for analytical runs and mixed with a rehydration solution to a total volume of 450 μl. Following rehydration for 14 h, IEF was performed with a low initial voltage (500–1,000 V) during the initial 2 h and then a voltage gradient of up to 8,000 V was applied with a limiting current of 15 μA/strip. The total product time × voltage applied was 69,920 vh for the analytical runs. The temperature was maintained at 20°C following IEF separation, and the gel strips were equilibrated for 2×15 min in an equilibration buffer containing 50 mmol/l Tris-HCl (pH 8.8), 6 M urea, 30% glycerol, 2% SDS and a trace of bromophenol blue. DTT (1%) was added to the first equilibration buffer and was replaced with 2.5% iodoacetamide in the second equilibration buffer. The equilibrated gel strips were then applied onto 1-mm thick 12.5% SDS linear polyacrylamide gradient vertical slab gels and sealed with 0.5% agarose. SDS-PAGE was run using Bio-Rad Protean II electrophoresis apparatus for 30 min at a constant current of 10 mA/gel and then switched to 25 mA/gel until the bromophenol blue frontier had reached the bottom of the gels. During the whole run, the temperature was set at 15°C. To determine the isoelectric point (pI) and molecular weight (Mr) of the separated proteins, 2-D standards were added to the protein samples as internal markers. Following 2-DE, the protein spots were visualized by a silver-based staining technique with the protein silver stain kit (Amersham Pharmacia Biosciences).

### Image analysis

The stained 2-DE gels were scanned using LabScan software on Imagescanner (Amersham Pharmacia Biotech). The spot intensity calibration, spot detection, background abstraction, matching, 1-D calibration and establishment of an average gel were performed using the ImageMaster 2D Elite 4.01 analysis software (Amersham Pharmacia Biotech). The intensity of each spot was quantified by calculating the spot volume following normalization of the image using the total spot volume normalization method, multiplied by the total area of all spots. The reproducibility of the spot position was calculated according to Gorbett’s method (16). The statistical analysis was performed with SPSS for Windows 10.0 and Excel (SPSS, Inc., Chicago, IL, USA).

### Enzymatic digestion of protein spots and MALDI-TOF-MS analysis of tryptic peptides

In-gel digestion was performed mainly according to the laboratory instructions. A total of 15 differential spots were excised from preparative gels using biopsy punches and then transferred to a 1.5-ml siliconized Eppendorf tube. One protein-free gel piece was treated in parallel as a negative control. The gel-spots were destained in a destaining solution consisting of 100 mmol/l Na_2_S_2_O_3_ and 30 mmol/l K_3_Fe(CN)_6_ (V/V, 1:1). The protein-containing gel-spots were reduced in a reduction buffer (100 mmol/l NH_4_HCO_3_ and 10 mmol/l DTT) for 1 h at 57°C and then in alkylation buffer (100 mmol/l NH_4_HCO_3_ and 55 mmol/l indoacetamide) in the dark for 30 min at room temperature. The gel pieces were dried in a vacuum centrifuge and then the dried gel-pieces were incubated in a digestion solution containing 50 mmol/l NH_4_HCO_3_, 5 Mm/l CaCl_2_ and 0.1 g/l TPCK-trypsin for 24 h at 37°C. The digest buffer was removed and saved. The gel pieces were then extracted with 100% ACN/5% TFA for 1 h at 37°C and the supernatant was removed. The extracts plus the first saved digest buffer were finally pooled and concentrated to 10 μl. The tryptic peptide mixture (1 μl) was mixed with 1 μl CCA matrix solution and vortexed gently. A volume (2 μl) of the mixture containing CCA matrix was loaded onto a stainless steel plate and air-dried, then 1 μl 0.1% TFA was added and removed after 30 sec, then air-dried. The sample was analyzed by Applied Biosystems Voyager System 4307 MALDI-TOF-MS (Applied Biosystems Inc.). The parameters were set up as follows: Positive ion-reflector mode; accelerating voltage, 20 KV; grid voltage, 64.5%; mirror voltage ratio, 1.12; N_2_ laser wavelength, 337 nm; pulse width, 3 nsec; number of laser shots, 50; acquisition mass range, 1,000–3,000 Da; delay, 100 nsec; and vacuum degree, 4×10^−7^ Torr. A trypsin-fragment peak served as an internal standard for mass calibration. A list of the corrected mass peaks formed the PMF.

### Database searching and identification of proteins

Proteins were identified using the PMF results by searching the SWISS-PROT/TrEMBL database (http://www.expasy.ch/sprot) using the PeptIdent software. The search parameters were set as follows: Mass tolerance, ±0.5 Da; number of missed cleavage sites, ≤1; cysteine residue modified as carbamidomethyl-cys; number of matched peptides, ≥4; species selected, *Homo sapiens* (human); peptide ion, [M+H]^+^; isotope masses were used; and the search range was within the experimental pI value of ±0.5 pH unit and the experimental Mr of ±20%.

### Data analysis

Data are expressed as the mean ± SD with the exception of the MTT and 2-DE data, which were expressed as the mean only. Data were analyzed using Student’s t-test. Statistical analysis was performed using SPSS for Windows 10.0 and Excel (SPSS, Inc.). P≤0.05 was considered to indicate a statistically significant difference.

## Results

### Result of 2-DE and ImageMast analysis

**Figure 1:**
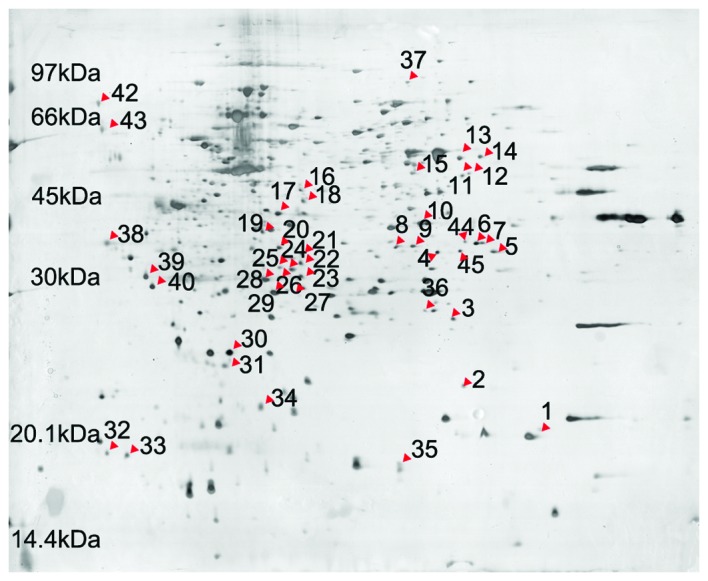
Two-dimensional gel electrophoresis (2-DE) profiles of untreated HCT116 cells. Red triangles represent differential protein-spots.

**Figure 2:**
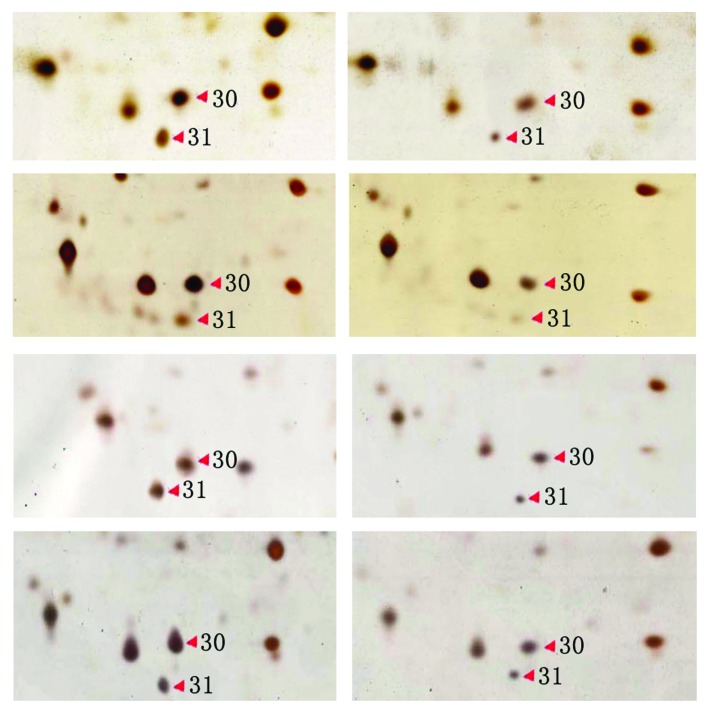
Partial two-dimensional gel electrophoresis (2-DE) profiles of indomethacin (IN)-treated and untreated HCT116 cells. There was decreased expression in IN-treated cells. Left, IN-untreated; right, IN-treated.

Using the same conditions and parameters, the experiment was repeated four times from cell culture to 2-DE, respectively. For the IN-treated and untreated HCT116 cells, a clear background and well-resolved and reproducible 2-DE patterns were attained. Fig. 1 shows 2-DE profiles of the untreated cells and Fig. 2 shows the partial 2-DE profiles of the IN-treated and untreated HCT116 cells at four different times. The results show that the levels of differentially-expressed protein spots 30 and 31 were decreased in the IN-treated group. Compared with the untreated maps, the average number of spots decreased by 7% in the IN-treated group (P<0.05). Furthermore, the differentially-detected protein spots between the IN-treated and untreated groups in the four experiments were consistent with a significant difference in relative volume (P<0.05) (Table I). Forty-five differential protein spots were identified between the IN-treated and untreated groups. Table I shows the relative volumes of the partial differential proteins.

### MALDI-TOF-MS PMF analysis of the differential protein spots

**Figure 3:**
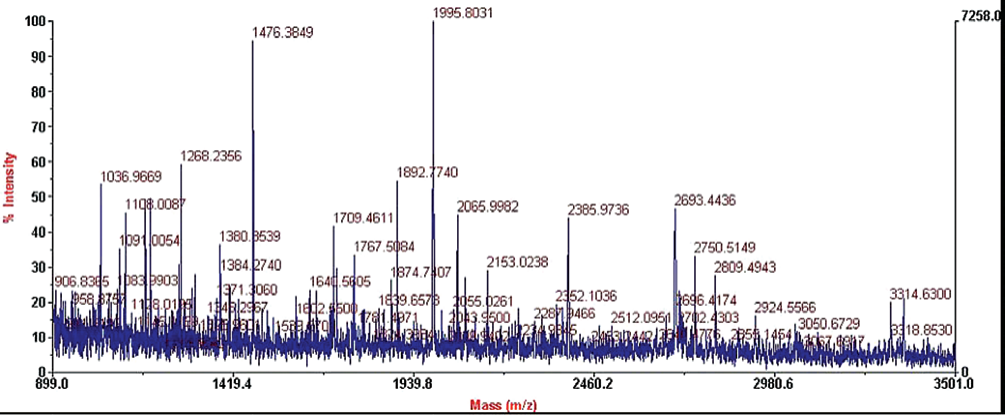
Peptide mass fingerprinting of differential protein spot 24 of IN-treated HCT116 cells. IN, indomethacin.

The differential protein spots between the IN-treated and untreated groups were detected by 2-DE gel image analysis software. To determine the accuracy of the matched result, two matched spots (16 differential protein spots in the IN-treated and untreated groups) were identified by MALDI-TOF-MS. The results showed that the two matched spots were the same protein [AC O95388, Wnt1-inducible signaling pathway protein 1 (WISP-1); Fig. 3]. Between ≥3 paired, IN-treated and untreated HCT116 cells, 15 differential protein spots were detected. These differential protein spots were excised from the silver stained gels and digested in-gel with trypsin. The PMF maps were obtained by MALDI-TOF-MS and calibrated with the TPCK-trypsin auto-degraded peak (m/z, 1,993.9772 Da). These PMF results were used to search the SWISS-PROT and TrEMBL databases using the PeptIdent software. The resulting proteins were determined by comprehensively considering the corresponding experimental pI, Mr, number of matched peptides and sequence coverage (Table I). Table II shows the matching of the differential protein spot 28 PMF results with protein Q03405 in the database, which identified a signaling pathway protein, WISP-1. It was first identified that HCT116 cells may be induced to undergo apoptosis by IN via the Wnt1 signaling pathway. Through the use of PMF, 15 differential proteins were identified, a number of which are the products of oncogenes and other molecules involved in the regulation of the cell cycle, apoptosis and signal transduction.

## Discussion

Compared with the genome, the proteome is dynamic, showing the various expression levels of proteins in various tissues and cells, the stages of up-growth and the physiology or pathology. The basic challenges of proteomics are identifying the proteins and predicting their functions. This is likely to lead to a new understanding of human biology, as well as to the design of new molecular structures as potential novel diagnostic or drug discovery targets (17). 2-DE is regarded as the most powerful separation method for resolving complex mixtures of proteins. Combined with mass spectrometry and ever-growing protein databases, powerful tools have become available that have made ‘functional proteomics’ feasible. Functional proteomics is defined as the use of proteomic methods to monitor and analyze molecular networks and fluxes within the living cell and to identify the molecular species that participate in these networks upon perturbation of the cellular environment. In order to further explore the molecular mechanisms of IN on CRC, a high-throughput proteomics technique was used in the current study to address the molecular basis of this effect by the study of the protein expression profiles of HCT116 cells prior to and following IN treatment. Analysis of the 2-DE profiles of the IN-treated and untreated cells was repeated four times. Quantitative expression changes were exhibited in 45 differential protein spots following IN-treatment, 34 of which decreased in abundance, 10 of which showed higher expression and 2 of which were expressed in untreated cells only. Differential protein spots (n=15) were selected to perform in-gel trypsin digestion and MALDI-TOF-MS-based PMF analysis, which identified a new signaling pathway protein, WISP-1. It was first identified that HCT116 cells may be induced to undergo apoptosis by IN via the Wnt1 signaling pathway. Specific proteins were shown to be the products of oncogenes and other molecules were involved in the regulation of the cell cycle, apoptosis and signal transduction.

The present study identified that IN induced apoptosis and inhibited the proliferation of HCT116 cells through various independent-COX methods. In addition, it was identified that HCT116 cells may be induced to undergo apoptosis by IN via the Wnt1 signaling pathway. Wnt family members are critical to a number of developmental processes, and components of the Wnt signaling pathway have been associated with tumorigenesis in familial and sporadic colon carcinomas (18). Wnt-1 is a member of an expanding family of cysteine-rich and glycosylated signaling proteins that mediate diverse developmental processes, including the control of cell proliferation, adhesion, cell polarity and the establishment of cell fates. It has been previously shown that WISP-1 activates the antiapoptotic Akt/PKB signaling pathway. It has also been demonstrated that WISP-1 prevents cells from undergoing apoptosis following DNA damage through the inhibition of the mitochondrial release of cytochrome *c* and the upregulation of antiapoptotic Bcl-X_L_(19). In the current study, the 24^th^ differential protein spot was identified as WISP-1, which decreased in expression in the IN-treated HCT116 cells. This implies that IN induces apoptosis and inhibits the proliferation of HCT116 cells.

In addition, the present study identified that the 30^th^ differential protein spot decreased in expression in the IN-treated cells. This spot was identified as Bcl-2-related protein A1 (BfL-1). The decrease in BfL-1 expression is likely to be an important mechanism to promote apoptosis in HCT116 cells. BfL-1 is a new member of the Bcl-2-related proteins, and previous studies have shown that the expression and regulation of Bcl-2 family members is one of the key factors of apoptosis. The Bcl-2 protein family is divided into antiapoptotic (e.g. BaX) and proapoptotic (e.g. Bcl-2 and Bcl-X_L_) proteins. Antiapoptotic Bax and proapoptotic Bcl-2 appear to form heterodimers, and the existence of Bcl-2/Bax heterodimers are responsible for the suppression and acceleration of apoptosis (20). It is known that members of the Bcl-2 family contain two conserved regions, Bcl-2 homology 1 and 2 (BH1 and BH2). Previous studies have shown that the deletion of BH1 and BH2 is important in order to form heterodimers with BAX. Overexpression of the Bcl-2 protein leads to Bcl-2/Bax heterodimers, which inhibit apoptosis (21,22). Thus, the ratio of Bcl-2 and Bax determines apoptosis sensitivity. Further investigations have shown (23) that NSAIDs may inhibit the expression of antiapoptotic protein Bcl-X_L_, resulting in an altered ratio of BAX to Bcl-X_L_ and subsequently, to mitochondria-mediated cell death. Chung *et al*(14) hypothesized that the overexpression of Bcl-2 or Bcl-x(L) prevents ROS production and the subsequent loss of mitochondrial membrane potential (Δψm), thereby, inhibiting apoptotic cell death. In the current study, IN induced the decreased expression of BfL-1, which may indicate the induced apoptosis of the HCT116 cells. Additionally, BfL-1 has two highly conserved regions known as BH1 and BH2 domains, which are necessary for the interaction between Bcl-2 or Bcl-X_L_ and BAX (22). It may be noteworthy to determine the additional BfL-1 heterodimers with BAX.

Mitogen-activated protein kinases (MAPK) are important signal transduction systems from extracellular signaling to intracellular reactions in eukaryotic cells. MAPK pathways are involved in a diverse set of responses affecting cell fate, including cell proliferation and differentiation, adaptation to environmental stress and apoptosis (24). MAPK cascades are key signaling pathways involved in the regulation of normal cell proliferation, survival and differentiation. The aberrant regulation of MAPK cascades contributes to cancer and other human diseases. The MAPKs are conserved proteins that regulate cell growth, division and death. To date, four types of MAPK pathways have been defined in mammalian cells. These include the extracellular signal regulated protein kinase pathway, the c-Jun NH_2_-terminal kinase/stress activated protein kinase pathway, the big MAPK/ERK5 pathway and the MAPK^p38^ pathway (25,26). The MAPK module includes three kinases that establish a sequential activation pathway comprising of MAPK kinase kinase (MKKK), MAPK kinase and MAPK. At present, it has been hypothesized that activation of the MAPK pathway contributes to cell hyperplasia and that the aberrant MAPK pathway contributes to apoptosis inhibition. The MAPK signal transduction pathway has been shown to be significant for the development of cell malignant transformation and tumor invasion and metastasis (27,28).

The current study identified that the 3^rd^ and 20^th^ differential protein spots exhibited decreased expression in IN-treated cells. These spots were identified as the Ras-related protein, Rab-39A, and MAPK3, p44 MAPK. The results show that the Ras-associated protein, Rab-39A, and the p44 MAPK protein are action targets of IN. Ligation of numerous receptors leads to the activation of MAPK^erk1/2^ through the activation of Ras. This was inferred from the observation that Ras activates MKKK^raf1^ and that activated MKKK^raf1^ is sufficient to stimulate the MAPK^erk1/2^ signaling pathway (24). It has been hypothesized that the activated p38 and p44 MAPK may direct ERKs into the cellular nucleus, which induces transcription factor phosphorylation, cell proliferation and differentiation. p44 MAPK is an isomer of ERK, named ERK1. Berger *et al*(29) showed that seven cell lines were noted to have mutations of K-ras, while seven cell lines did not. No difference in the expression of Raf-1 was identified between the K-ras mutant and non-mutant cell lines. However, there was a significant increase in MAPK activity in the non-mutant cell lines compared with the cell lines with Ras mutations (P=0.026). This may be associated with the active expression of Raf-1 in these cell lines. Schwenger *et al*(30) hypothesized that IN fails to induce a significant activation of p38 MAPK, but that salicylates induce p38 MAPK activation. In the current study, the p44 MAPK protein was downregulated in the IN-treated cells. The results showed that IN inhibited the proliferation of the HCT116 cells through the inhibited expression of the p44 MAPK protein in the MAPK signaling pathway. These results are consistent with the inhibited proliferation of pancreatic tumor cells with NSAID therapy via the MEK-ERK signaling pathway (31).

Overall, the use of functional proteomics in the present study showed that IN may induce apoptosis and inhibit the proliferation of HCT116 cells by independent-COX. This confirms a new method to study the antitumor mechanisms of IN.

## Figures and Tables

**Table I:** Characterized differential expression of IN-treated and untreated HCT116 cells.

Protein spot no.	Peptide matches, n	AC	Protein description	Coverage, %	Theoretical molar mass, KDa/pI	Experimental molar mass, KDa/pI	Relative volume of untreated, %	Relative volume of IN-treated, %	P-value
3	5/50	Q14964	Ras-related protein Rab-39	30.0	24.87/6.90	27.82/7.61	0.238±0.043	0.136±0.041	0.043
4	9/30	Q92782	Zinc finger protein neuro-d4	45.5	38.91/6.28	38.14/7.44	0.236±0.019	0.077±0.027	0.027
5	6/37	Q9BQ16–2	Splice isoform 2 of testican-3 precursor	41.2	35.59/8.79	38.30/8.08	0.319±0.029	0.122±0.035	0.034
6	8/57	P00747	Angiostatin	40.2	41.64/7.74	40.13/7.87	0.334±0.037	0.193±0.007	0.026
8	9/53	O14753	Putative transcription factor Ovo-like1	37.4	Undefined	39.32/6.98	0.236±0.014	0.071±0.012	0.017
9	11/60	P48745	Chain1:NOV protein homolog	35.8	36.09/7.95	39.54/7.17	0.233±0.023	0.070±0.014	0.010
11	20/76	Q9Y297–2	Splice insoform 2 of F-box/ WD-repeat protein 1A	30.1	65.05/8.24	56.08/7.72	0.227±0.040	0.065±0.027	0.006
14	11/59	Q9H7B4	Set and Mynd domain containing protein 3	31.5	49.11/6.75	58.02/7.92	0.225±0.006	0.017±0.001	0.001
20	8/40	P27361	p44MAPK	26.8	43.74/6.28	39.16/5.66	0.234±0.054	0.080±0.029	0.017
24	12/34	O95388	WISP-1	36.5	38.01/6.47	34.34/5.80	0.320±0.011	0.128±0.015	0.001
28	9/20	Q03405	uPAR	83.2	31.46/5.95	32.04/5.51	0.404±0.021	0.249±0.036	0.049
30	4/35	Q16548	Bfl-1	32.6	20.13/5.32	25.37/5.10	0.516±0.107	0.093±0.072	0.039
31	5/51	P12004	PCNA	25.9	28.77/4.57	24.43/5.04	0.293±0.009	0.077±0.016	0.002
36	4/34	O43609	spry-1	40.3	Undefined	28.44/7.29	0.070±0.003	0.197±0.012	0.007
39	5/36	P17034	Zinc finger protein KOX23	87.5	Undefined	32.79/4.19	0.343±0.042	0.512±0.063	0.029

Relative volumes presented as mean ± standard deviation. IN, indomethacin; pI, isoelectric point; WISP-1, Wnt1-inducible singnaling pathway protein 1; uPAR, urokinase plasminogen activator receptor; Bfl-1, Bcl-2 related protein A1; PCNA, proliferating cell nuclear antigen; spry-1, sprouty homolog 1; p44 MAPK, mitogen-activated protein kinase 3; AC, accession number.

**Table II:** Matching of differential protein spot 28 PMF results with protein Q03405 in the database.

User mass(Da)	Matching mass (Da)	Δ mass (Da)	MC	Modification	Position	Peptide
1274.2457	1274.4745	0.2288	1	1xMSO	25–35	CMQCKTNGDCR
1445.2916	1445.5389	0.2473	1	3xCys_CAM, 1xMSO	25–35	CMQCKTNGDCR
1995.8514	1995.7915	−0.0598	0	3xCys_PAM	114–129	YLECISCGSSDMSCER
1995.8514	1995.8027	−0.0486	1		114–131	YLECISCGSSDMSCERGR
2679.0663	2679.2324	0.1661	1	1xCys_PAM	198–220	CNEGPILELENLPQNGRQCYSCK
2692.4470	2692.3393	−0.1076	1	2xCys_CAM	81–105	TGLKITSLTEVVCGLDLCNQGNSGR
2750.4127	2750.2695	−0.1431	1	2xCys_PAM	198–220	CNEGPILELENLPQNGRQCYSCK
2810.3884	2810.1637	−0.2246	1	2xCys_PAM	215–238	QCYSCKGNSTHGCSSEETFLIDCR
3123.8803	3123.4436	−0.4366	1	Cys_PAM:144	139–164	SPEEQCLDVVTHWIQEGEEGRPKDDR
3124.9551	3125.2825	0.3274	0	1xCys_PAM, 1xMSO	262–290	GCATASMCQHAHLGDAFSMNHIDVSCCTK

PMF, peptide mass fingerprint; MC, missed cleavages.

## Abbreviations

NSAIDs: non-steroidal anti-inflammatory drugs
COX: cyclooxygenase
2-DE: two-dimensional gel electrophoresis
PMF: peptide mass fingerprint
IN: indomethacin
MALDI-TOF-MS: matrix-assisted laser desorption/ionization time of flight mass spectrometry
